# Unassigned MURF1 of kinetoplastids codes for NADH dehydrogenase subunit 2

**DOI:** 10.1186/1471-2164-9-455

**Published:** 2008-10-02

**Authors:** Sivakumar Kannan, Gertraud Burger

**Affiliations:** 1 Robert Cedergren Research Center for Bioinformatics and Genomics, Département de Biochimie, Université de Montréal, 2900 Boulevard Edouard-Montpetit, Montréal, Québec, H3T 1J4, Canada

## Abstract

**Background:**

In a previous study, we conducted a large-scale similarity-free function prediction of mitochondrion-encoded hypothetical proteins, by which the hypothetical gene *murf1 *(maxicircle unidentified reading frame 1) was assigned as *nad2*, encoding subunit 2 of NADH dehydrogenase (Complex I of the respiratory chain). This hypothetical gene occurs in the mitochondrial genome of kinetoplastids, a group of unicellular eukaryotes including the causative agents of African sleeping sickness and leishmaniasis. In the present study, we test this assignment by using bioinformatics methods that are highly sensitive in identifying remote homologs and confront the prediction with available biological knowledge.

**Results:**

Comparison of MURF1 profile Hidden Markov Model (HMM) against function-known profile HMMs in Pfam, Panther and TIGR shows that MURF1 is a Complex I protein, but without specifying the exact subunit. Therefore, we constructed profile HMMs for each individual subunit, using all available sequences clustered at various identity thresholds. HMM-HMM comparison of these individual NADH subunits against MURF1 clearly identifies this hypothetical protein as NAD2. Further, we collected the relevant experimental information about kinetoplastids, which provides additional evidence in support of this prediction.

**Conclusion:**

Our *in silico *analyses provide convincing evidence for MURF1 being a highly divergent member of NAD2.

## Background

The single-celled flagellated eukaryotes of the group kinetoplastids include notorious human pathogens such as *Trypanosoma *and *Leishmania*. Mitochondrial (mt) genomes of numerous trypanosomatids have been sequenced, with complete and nearly complete mtDNA sequences available for five species: *Leishmania tarentolae *(GenBank Accession No: NC_000894), *Trypanosoma brucei *(M94286), *T. cruzi *(DQ343645), *Crithidia oncopelti *(X56015), *Leptomonas seymouri *(DQ239758), and major portions of mtDNA for two other members of the group: *Leishmania major *(AH015294), *Leptomonas collosoma *(AH015822). For a review, see [1].

The unassigned open reading frame (ORF) *murf1 *in *T. brucei *mtDNA has been known for 25 years, but until today, there is no protein of known function that shares significant sequence similarity with this ORF [2]. In a recent study, we conducted a comprehensive function prediction of all hypothetical mitochondrion-encoded proteins using a machine-learning-based classifier MOPS [3]. This classifier does not rely on sequence similarity but rather on a host of other features including physico-chemical properties of proteins, and hence should be able to detect remote homologs. MOPS predicted, but only with moderate support, MURF1 of the kinetoplastid *Phytomonas serpens *as subunit 2 (NAD2) of the NADH-Ubiquinone Oxidoreductase (NADHdh) or Complex I of the electron transport chain – a multi-complex pathway embedded in the inner mitochondrial membrane. NADHdh is the largest complex of this pathway with ~45 distinct subunits, seven of which are usually encoded in the mitochondria. We chose to scrutinize this function assignment in detail, motivated by several reasons: the long-standing controversy surrounding MURF1, the large available body of related biological knowledge, and the significance of this organismal group for human health [2,4-6].

## Results

As mentioned in the Background, the hypothetical protein MURF1 was predicted by the automated similarity-free classifier MOPS to be a divergent NADHdh subunit 2 (NAD2). To test this prediction, we conducted the following analyses.

### Sequence – Sequence Comparison

BLAST searches of *Phytomonas *MURF1 sequence against NRDB or UniProt did not result in any informative hits, but identified all the MURF1 homologs from other kinetoplastids such as *T. brucei, L. tarentolae*, etc. In contrast, FASTA searches against UniProt returned, after MURF1 homologs, NADHdh subunit 5 from the kinetoplastid *Crithidia *as top informative hit with an e-value of 6.5e^-09^, followed by NAD2 from the red alga *Chondrus crispus *with an e-value of 8.8e^-07^. A list of all hits and their corresponding e-values is compiled in Table 1.

**Table 1:** List of FASTA hits for *P. serpens *MURF1 searched against UniProt

**UniProt ID**	**Species Name**	**Protein Name**	**e-value**	**Similarity**
Q9XKY50	*Phytomonas serpens*	MURF1	5.3e^-148^	100.0%
Q33559	*Leishmania tarentolae*	MURF1	2.7e^-109^	90.9%
Q8HE85	*Trypanosoma sp.*	MURF1	3.1e^-17^	87.6%
Q33547	*Blastocrithidia culicis*	MURF1	1.2e^-16^	86.5%
Q33552	*Crithidia fasciculata*	MURF1	6e^-16^	88.4%
Q33556	*Herpetomonas muscarum*	MURF1	5.1e^-13^	85.0%
Q34937	*Leishmania. tarentolae*	MURF2	2e^-09^	60.4%
Q34096	*Crithidia fasciculata*	MURF2	3.2e^-09^	56.3%
Q34192	*Crithidia oncopelti*	NAD5	3.8e^-09^	54.5%
P48903	*Chondrus crispus*	NAD2	5.4e^-07^	57.9%
Q5LRX2	*Silicibacter pomeroyi*	Putative membrane protein	1.2e^-06^	58.0%
Q6E773	*Saprolegnia ferax*	NAD2	1.5e^-06^	53.8%
Q6SKY5	*Speleonectes tulumensis*	NAD5	2.3e^-06^	55.2%
Q5AG49	*Candida albicans*	Hypothetical protein	3.3e^-06^	67.7%
Q5AGI5	*Candida albicans*	Hypothetical protein	7.1e^-06^	67.9%
Q8SKS6	*Ancylostoma duodenale*	NAD4	7.4e^-06^	57.3%
Q85TH7	*Melipona bicolor*	NAD4	7.7e^-06^	58.1%
Q33575	*Trypanosoma brucei*	NAD4	8.7e^-06^	57.3%
P24499	*Trypanosoma brucei brucei*	ATP6	1.1e^-05^	55.4%
Q70NW4	*Strongyloides stercoralis*	NAD4	1.2e^-05^	56.7%
Q33570	*Trypanosoma cruzi*	ATP6	1.5e^-05^	56.9%
Q5CV17	*Cryptosporidium parvum*	Hypothetical protein	1.5e^-05^	61.5%
Q057W5	*Buchnera aphidicola*	NADH dehydrogenase I chain L	1.9e^-05^	54.9%
Q8IBJ6	*Plasmodium falciparum*	Hypothetical protein	2.9e^-05^	58.4%

### Profile – Sequence Comparison

For the identification of distantly related sequences, methods that exploit profiles (i.e., position-specific descriptions of the consensus of a multiple sequence alignment) are more sensitive than those based on pairwise alignment such as BLAST and FASTA. Here, we used PSI-BLAST to generate a MURF1 profile and searched it against NRDB, but no other proteins beyond kinetoplastid MURF1 sequences were found.

### Profile HMM – Profile HMM Comparison

Our hypothesis is that MURF1 is a highly derived distant homolog of NAD2. We used Profile HMM – Profile HMM comparison because it is the most sensitive method in identifying distant homologs. In contrast to simple sequence profiles, Profile Hidden Markov Models (HMMs) contain extra information about insertions/deletions and gap scores. HHsearch (the first implementation of this approach), was shown to outperform profile – sequence comparison methods such as PSI-BLAST and HMMER, profile – profile comparison tools such as PROF_SIM and COMPASS and the other HMM – HMM comparison tool PRC [7].

We built a profile HMM for MURF1 from the multiple alignment of several kinetoplastid MURF1 sequences. Using HHsearch, we searched this profile HMM against the profile HMMs available in Pfam, PANTHER, COG and TIGR. In most cases, the top hit was to the "NADH-Ubiquinone/plastoquinone (Complex I)" profile HMM, which was built from 12 distinct subunits of different function. Though these subunits are non-homologous proteins, Pfam puts them all together in to a single family because they share high hydrophobicity (transmembrane domains). Only the search against the COG database returned a specific subunit as top hit, i.e., NAD2. HHsearch results are summarized in Table 2.

**Table 2:** Best informative hits for the MURF1 profile HMM when searched against profile HMMs from various databases

	**Best informative hit**	**e-value**	**Identity**	**Probability**
Pfam	NADH-Ubiquinone/plastoquinone (Complex I), various subunits	1.6e^-08^	21%	96.80
PANTHER	NADH dehydrogenase	4.3e^-09^	16%	99.20
COG	NADH:Ubiquinone oxidoreductase subunit 2	3.8e^-03^	19%	39.65
TIGR	NDH_I_N Proton-translocating NADH-Quinone oxidoreductase	91	19%	75.95

To narrow down the exact function of MURF1, we generated profile HMMs for all 12 subunits of NADHdh. For that, we clustered the protein sequences of all NADHdh subunits at different identity thresholds ranging from 40% to 75%, constructed a multiple sequence alignment for each of the subunits at each threshold, and generated a total of 84 profile HMMs. We then searched the MURF1 profile HMM against all the profiles of NADHdh subunits. As expected for remote homologs, the scores are relatively low. The six top hits are NAD2 with an e-values ranging from 2.70e^-15 ^to 1e^-11^. The e-value of the other subunit best hits is 4 orders of magnitude worse (Table 3).

**Table 3:** Best hits for the MURF1 profile HMM when searched against the profile HMMs of all NADH dehydrogenase subunits using HHsearch. The hits are ranked based on E-values^a^

**No**	**Hit^b^**	**Probability**	**E-value**	**Identities**	**Score**
1	NAD2_0.45	96.6	2.70E-15	26	75.7
2	NAD2_0.4	96.6	3.20E-15	25	75.3
3	NAD2_0.5	96.6	1.70E-14	23	72.1
4	NAD2_0.55	96.5	1.50E-12	34	63.1
5	NAD2_0.6	96.3	6.30E-12	23	60.4
6	NAD2_0.65	96.2	1.00E-11	26	59.4
7	NAD4_0.4	96	2.10E-11	21	58
8	NAD4_0.55	95.9	2.90E-11	23	57.4
9	NAD4_0.6	95.2	1.40E-10	28	54.2
10	NAD2_0.7	95.1	1.90E-10	27	53.6
11	NAD4_0.7	95	2.40E-10	28	53.2
12	NAD4_0.5	94.3	6.00E-10	28	51.4
13	NAD2_0.75	93.8	1.20E-09	24	50
14	NAD4_0.45	93.2	2.00E-09	27	49
15	NAD4_0.75	93.1	2.30E-09	28	48.7
16	NAD6_0.4	91.8	6.40E-09	24	46.7
17	NAD6_0.45	90.1	1.80E-08	26	44.7
18	NAD5_0.4	89.8	2.10E-08	21	44.4
19	NAD1_0.55	88.9	3.30E-08	18	43.5
20	NAD6_0.5	88.9	3.30E-08	23	43.5
21	NAD5_0.5	88.4	4.00E-08	28	43.1
22	NAD1_0.6	86.7	8.10E-08	17	41.7
23	NAD1_0.5	86	1.10E-07	18	41.2
24	NAD6_0.55	85.7	1.20E-07	25	41
25	NAD5_0.55	85.7	1.20E-07	25	40.9
26	NAD1_0.65	84.8	1.60E-07	20	40.4
27	NAD1_0.4	84.3	1.80E-07	24	40.1
28	NAD1_0.45	84.1	2.00E-07	22	40
29	NAD4_0.65	83.5	2.40E-07	26	39.6
30	NAD1_0.7	83.2	2.60E-07	21	39.4
31	NAD5_0.45	25	2.90E-07	25	39.2
32	NAD5_0.6	80.4	5.50E-07	21	37.9
33	NAD6_0.65	80	6.10E-07	28	37.7
34	NAD1_0.75	79.5	6.90E-07	18	37.5
35	NAD5_0.65	77.3	1.10E-06	18	36.5
36	NAD5_0.75	76.9	1.20E-06	21	36.3
37	NAD5_0.7	76.7	1.30E-06	18	36.2
38	NAD6_0.6	73.6	2.40E-06	25	35
39	NAD6_0.7	69.8	4.70E-06	25	33.7
40	NAD6_0.75	69.2	5.20E-06	20	33.5
41	NAD3_0.4	62.1	1.50E-05	30	31.4
42	NAD3_0.45	55.9	3.50E-05	27	29.8
43	NAD3_0.55	48.2	8.80E-05	25	27.9
44	NAD3_0.6	46.9	0.0001	24	27.6
45	NAD3_0.65	45.8	0.00012	23	27.4
46	NAD3_0.5	43.9	0.00014	21	27
47	NAD4L_0.4	34.2	0.00044	25	24.8
48	NAD4L_0.45	31.2	0.00062	18	24.1
49	NAD4L_0.55	26.8	0.0011	15	23
50	NAD3_0.7	26.7	0.0011	26	23
51	NAD4L_0.6	26.5	0.0011	30	22.9
52	NAD4L_0.7	23.3	0.0016	27	22.1
53	NAD4L_0.5	21.1	0.0022	18	21.6

^a ^Probability, e-value, identity and score for each hit were reported by HHSearch

^b ^The number following the subunit name is the sequence identity threshold used for clustering the sequences from which we generate the profile HMM. For example, NAD2_0.45 profile HMM is generated by clustering all known NAD2 sequences at 45% sequence identity threshold using CD-HIT.

## Discussion

While sequence – sequence comparison and profile HMM – profile HMM comparison point to MURF1 being a subunit of NADHdh, profile – profile comparison against the profile HMMs of individual subunits of NADHdh is able to clearly assign MURF1 to NAD2. In the following, we will confront this *in silico *prediction with the available biological knowledge. If the MURF1 protein of trypanosomes is indeed NAD2, then the following criteria must apply.

**1. There should be no previously annotated *nad*2 gene in either mitochondrial or nuclear genomes of kinetoplastids**. A *nad*2 gene has not been reported in any mitochondrial genome of kinetoplastids. Recently, the sequence of the nuclear genome became available for the *P. serpens *[4]. Neither genome nor EST data (2,190 ESTs) indicate the presence of this gene.

**2. There should be numerous precedents for *nad*2 being encoded by mtDNA**. The *nad*2 gene is mtDNA-encoded by the large majority of eukaryotes (see GOBASE, 'Gene Distribution' ). The rare species that lack this mitochondrial gene also lack other NADH subunits (Apicomplexa, yeast).

**3. The *murf1 *gene should be transcribed**. Evidence for *murf1 *being expressed rather than being a spurious ORF is provided by several observations. First, the deduced amino acid sequence is conserved across trypanosomes, despite considerable divergence at the nucleotide level. Second, transcription of this gene has been demonstrated in *P. serpens *[5].

**4. Rotenone-sensitive NADH dehydrogenase Complex I should be present in kinetoplastids**. The presence of Complex I has been biochemically confirmed in *Trypanosoma *and *Phytomonas *[6,8].

## Conclusion

**Figure 1:**
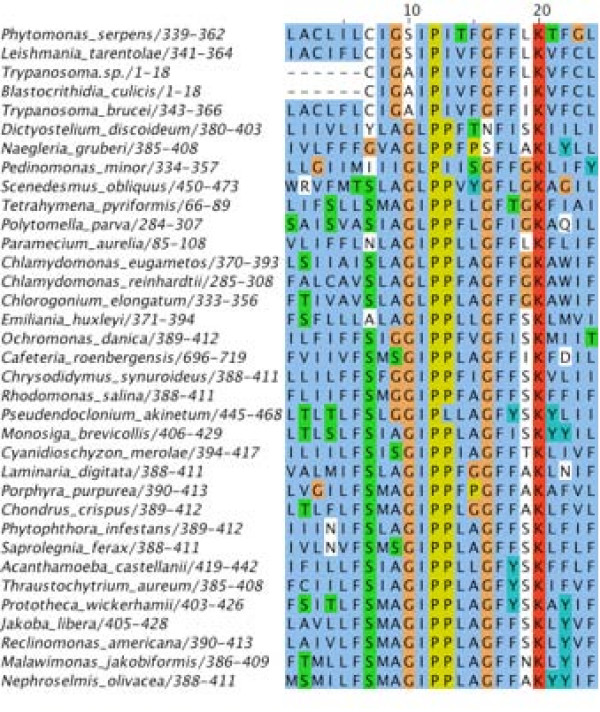
**Multiple sequence alignment of kinetoplastid MURF1 sequences with NAD2 sequences from other eukaryotes. **The top five sequences are kinetoplastid proteins. Only the most conserved region of the protein is depicted. The range of amino acid positions included in the alignment is indicated by the numbers following the species name. Dashes specify alignment gaps.

On all accounts enumerated above, the biological knowledge reinforces the *in silico *prediction. Together, this provides convincing evidence that MURF1 is a highly derived homolog of NAD2. For illustration purpose, Fig. 1 depicts the multiple protein sequence alignment of the most conserved block of known NAD2 proteins and kinetoplastid MURF1 sequences.

### Outlook

Notably, a functional NADHdh is crucial to the survival of trypanosomes. Under aerobic conditions (procyclic, insect stage), NADHdh is required as a component of the respiratory chain, to catalyze electron transport toward complex IV. The thus generated proton gradient is utilized for ATP synthesis. Under anaerobic conditions (bloodstream form), a functional NADHdh is equally essential. In the blood stream of mammals, NADHdh provides electrons for the alternative oxidase, a pathway required for maintaining the balance of NADH/NAD+ in the cell. This confirms that trypanosomes depend on a functional NADHdh. In fact, Atovaquone, an anti-leishmanial drug, inhibits the NADHdh activity in *P. serpens *and this inhibition was suggested to underlie the anti-leishmanial activity of that drug [6]. In this context, the identification of MURF1 as a divergent NAD2 could offer new avenues to the prevention or treatment of trypanosomatid-caused diseases.

## Methods

### Dataset

All function-known protein sequences used in this study were retrieved from the organelle genome database GOBASE release 12.0 [9]. The homologs for MURF1 were retrieved from Entrez, and their accession numbers are given in Table 4[10].

**Table 4:** List of kinetoplastid MURF1 sequences with GenBank Accession Numbers

**Species Name**	**GenBank Accession**
*Phytomonas serpens*	AAD28358
*Leishmania tarentolae*	NP_050068
*Trypanosoma brucei*	E22845
*Trypanosoma sp.*	AAN86606
*Blastocrithidia culicis*	AAA73417
*Crithidia fasciculata*	AAA73421
*Herpetomonas muscarum*	AAA73415

### Assignment of MURF1

For the function assignment of MURF1, we chose to use sequence-sequence, sequence-profile and profile-profile methods described below, which are most sensitive methods to detect remote homologs.

### Sequence – Sequence Comparison

A BLAST (blastp) search was conducted for the MURF1 protein sequence against NCBI's NRDB (non-redundant protein database) (October, 2006; 4,565,699 sequences), with default parameters [11]. In addition, a FASTA search was conducted for the MURF 1 protein sequence against UniProt (release 10.4) with default parameters, at the EBI website [12].

### Profile – Sequence Comparison

This comparison was conducted in two different ways. First, PSI-BLAST was employed to search MURF1 remotely against NCBI's NRDB, with four iterations [13]. Second, we performed profile HMM – sequence comparison using profiles from Pfam version 21.0, executed at the Pfam website [14].

### Profile HMM – Profile HMM Comparison

For Profile HMM – profile HMM comparison, we used HHsearch of the HHpred package, which takes the MURF1 sequence as input and searches against NRDB using PSI-BLAST [15]. The MURF1 homologs obtained from the PSI-BLAST search are then used to generate a profile HMM. As a next step, this MURF1 profile HMM is searched against all profile HMMs of function-known proteins available from the public databases Pfam, PANTHER, SMART, COG, PDB and SCOP.

In addition, we generated our own profile HMMs for each of the 12 NADHdh subunits (1–11 and 4L) from all known sequences of these protein classes. These sequences were clustered at eight different identity thresholds (40, 45, 50, 55, 60, 65, 70 and 75%) using CD-HIT, followed by multiple sequence alignment performed with MUSCLE [16,17]. (Note: The number of instances for subunit NAD8 and NAD10 are less than 3 at identity thresholds 65 and 75% respectively and hence profile HMMs were not generated below these thresholds for these two subunits) [see Additional file 1]. The multiple alignment served as input for generating profiles using hmmbuild of HMMER version 2.3.2, 2003 package [18]. In order to verify whether profile HMM-profile HMM comparison is efficient in distinguishing the subunits, we tested this approach on the function-known sequences. Herefore, we used NAD2 and NAD5 subunits – the most difficult subunits to distinguish. For evaluating NAD2-profile HMMs, all NAD2 sequences were divided randomly into ten non-overlapping subsets of equal size. A test-profile HMM was generated using one of the subsets, while the remaining nine subsets were used for generating a "master" profile HMM. The NAD2 test-profile HMM was then searched against the NAD2 "master" profile HMM and the NAD5 profile HMM (generated using all NAD5 sequences) using HHsearch. This procedure is repeated ten times. The same test was done for NAD5. All test-profile HMMs were correctly identified at 100%. Finally, the MURF1 profile HMM was searched against all the 84 profiles using HHsearch with default parameters.

## Authors' contributions

SK carried out the sequence analyses and drafted the manuscript. GB conceived the study, supervised and helped to draft the manuscript. Both authors read and approved the final manuscript.

## Supplementary Material

Additional file 1 **Number of NADHdh subunit sequences after clustering at different identity thresholds.** This table shows the number of various NADHdh subunit sequences obtained after clustering at identity thresholds from 99 – 40% using CD-HIT. Click here for file
