# Supplementary material for: Unassigned MURF1 of kinetoplastids codes for NADH dehydrogenase subunit 2
Source: BMC Genomics. 2008 Oct 2;9:455. doi: 10.1186/1471-2164-9-455 (PMC2572627; doi:10.1186/1471-2164-9-455)
Supplement: Additional file 1 — Number of NADHdh subunit sequences after clustering at different identity thresholds. This table shows the number of various NADHdh subunit sequences obtained after clustering at identity thresholds from 99 – 40% using CD-HIT. [file 1471-2164-9-455-S1.pdf]

Additional File 1. Number of NADHdh subunit sequences after clustering at different identity thresholds

	**99%**	**75%**	**70%**	**65%**	**60%**	**55%**	**50%**	**45%**	**40%**
NAD1	1538	293	234	196	150	112	76	57	40
NAD2	4038	580	518	396	292	243	202	162	123
NAD3	1190	332	260	220	169	130	105	76	49
NAD4	1118	328	267	206	171	138	111	74	52
NAD4L	1003	333	275	221	195	160	126	104	86
NAD5	1010	399	312	249	199	158	128	87	54
NAD6	1061	500	431	373	314	265	232	183	145
NAD7	37	15	11	7	6	4	4	4	4
NAD8	5	4	4	2	2	2	2	2	2
NAD9	45	25	23	22	20	19	14	13	9
NAD10	7	2	2	2	1	1	1	1	1
NAD11	18	15	14	14	12	11	9	9	6
